# The costs and potential savings of a novel telepaediatric service in Queensland

**DOI:** 10.1186/1472-6963-7-35

**Published:** 2007-03-02

**Authors:** Anthony C Smith, Paul Scuffham, Richard Wootton

**Affiliations:** 1 Centre for Online Health, University of Queensland, Level 3 Foundation Building, Royal Children's Hospital, Herston, Brisbane, Queensland, 4029, Australia; 2 School of Medicine, Griffith University, Logan Campus, Brisbane, Queensland, Australia

## Abstract

**Background:**

There are few cost-minimisation studies in telemedicine. We have compared the actual costs of providing a telepaediatric service to the potential costs if patients had travelled to see the specialist in person.

**Methods:**

In November 2000, we established a novel telepaediatric service for selected regional hospitals in Queensland. Instead of transferring patients to Brisbane, the majority of referrals to specialists in Brisbane were dealt with via videoconference. Since the service began, 1499 consultations have been conducted for a broad range of paediatric sub-specialities including burns, cardiology, child development, dermatology, diabetes, endocrinology, gastroenterology, nephrology, neurology, oncology, orthopaedics, paediatric surgery and psychiatry.

**Results:**

During a five year period, the total cost of providing 1499 consultations through the telepaediatric service was A$955,996. The estimated potential cost of providing an outpatient service to the same number of patients at the Royal Children's Hospital in Brisbane was A$1,553,264; thus, telepaediatric services resulted in a net saving of approximately A$600,000 to the health service provider.

**Conclusion:**

Telepaediatrics was a cheaper method for the delivery of outpatient services when the workload exceeded 774 consultations. A sensitivity analysis showed that the threshold point was most sensitive to changes related to patient travel costs, coordinator salaries and videoconference equipment costs. The study showed substantial savings for the health department, mainly due to reduced costs associated with patient travel.

## Background

In terms of area, Queensland is the second largest state in Australia with a land area of about 1,722,000 km^2 ^[1]. The estimated population of Queensland is four million people [2]. Because the majority of specialist services are located in the south-eastern corner of Queensland, people living elsewhere may have to travel up to 2500 km to see a specialist. This usually requires hours of driving by car or expensive journeys by rail or air.

The health department in Queensland offers a subsidy scheme to patients in regional and remote areas. That is, if a patient in a regional or remote area of Queensland needs to travel to see the specialist, the majority of the travel and accommodation costs are reimbursed by the state government. The total cost is approximately A$30 M per year [3].

We have evaluated a novel telepaediatric service in Queensland over a five year period. The service provides a centralised call centre to selected paediatricians in regional sites throughout the state [4]. If a specialist consultation is required, the referring paediatrician directs the referral through the call centre to a telepaediatric coordinator. The coordinator takes responsibility for each referral and provides a guaranteed response by an appropriate specialist. The majority (85%) of all responses facilitated by the telepaediatric service involve a videoconference. The remaining cases are dealt with via telephone or email.

The telepaediatric service offers a broad range of paediatric sub-specialties including burns, cardiology, child development, dermatology, diabetes, endocrinology, gastroenterology, nephrology, neurology, oncology, orthopaedics, paediatric surgery and psychiatry [5-8]. The service gives families the opportunity for a specialist consultation, without the need for extensive travel to Brisbane. The saved journeys to Brisbane result in direct benefits to the family, including fewer out-of-pocket expenses and reduced stress and inconvenience associated with time away from home [9].

In the present study, we examined the costs of providing the telepaediatric service to two regional hospitals in Queensland – at Mackay and Hervey Bay – over a five year period. The hospitals are 1100 km and 350 km north of Brisbane respectively. We compared these costs with the potential costs had patients travelled to Brisbane to see the specialist in person (conventional outpatients).

## Methods

Telepaediatric service records were reviewed to obtain the number of consultations, types of specialist services offered, videoconference time spent and origin of referrals. We compared the actual costs of providing telepaediatrics to the potential costs if patients travelled to the Royal Children's Hospital (RCH) for an outpatient appointment.

The fixed and variable costs for both services were determined. The costs of operating the telepaediatric service included the purchase of videoconference equipment, salaries for the coordinators, salaries for clinical staff, telecommunication charges and miscellaneous project expenses.

The main costs associated with the transfer of patients to Brisbane for an outpatient appointment at the RCH included staff salaries, patient travel and accommodation. All costs are described in Australian Dollars ($A) at 2005 prices and include 10% Goods and Services Tax (GST); A$1 = approximately US$0.75. All unit costs were rounded to the nearest dollar value unless otherwise specified.

The equipment was three videoconference systems, two computers and one printer. The capital cost of these items was converted into an annual equivalent cost through the process of annuitizing these costs over the expected life of the equipment (5 y) using an annual discount rate of 5% [10]; this annual equivalent cost was then multiplied by the duration of the trial to calculate the attributable cost. The equipment was assumed (conservatively) to have no resale value after the five year period.

Actual travel costs were provided by the hospital patient travel department for patients who did travel to the RCH. Costs used in this analysis were calculated by determining the average costs of travel, doubling the average cost per fare (i.e. making allowance for one parent to accompany the child) and multiplying this cost by the number of consultations that took place from each site. Accommodation costs assumed that 10% of families travelling to Brisbane would require at least one night's accommodation before returning home [11].

The average and variable cost per consultation were determined for both services. The average cost was the total service cost divided by the number of consultations. The variable cost per consultation was the total variable cost divided by the number of consultations.

The threshold was calculated at which the cost of providing telepaediatric consultations equalled the cost of providing the same service in the conventional manner, i.e. face to face [11]. This was:

Total cost of telepaediatrics = Total cost of RCH outpatients

F_*vc *_+ *x*V_*vc *_= F_*ftf *_+ *x*V_*ftf*_

*x *= (F_*vc *_- F_*ftf*_)/(V_*ftf *_- V_*vc*_)

*x *= number of consultations; F = Fixed costs; V = Variable cost; *ftf *= Face to face *vc *= Videoconference;

A sensitivity analysis was conducted to investigate the effects on the threshold point of the assumptions made in the analysis. Thus the sensitivity could be used to identify those costs that had the greatest effect on the threshold, and those costs which had the least effect. Permission to conduct this research was granted by the RCH Ethics Committee and the RCH Executive Management Committee.

## Results

### Telepaediatric activity

**Figure 1:**
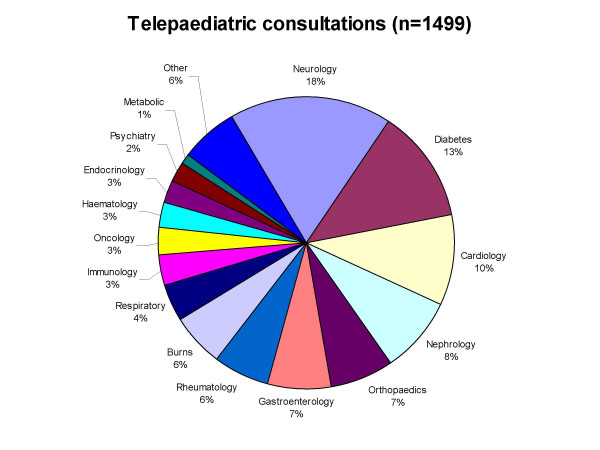
Telepaediatric activity – Mackay and Hervey Bay (n = 1499 consultations).

In the five year period from November 2000 to October 2005, 1499 consultations were conducted for patients at the two sites. 1139 (76%) of the consultations took place in Mackay, whilst the remainder (360) involved Hervey Bay. A total of 545 h was spent providing clinical consultations via videoconference. Consultations involved medical, nursing and allied health staff for 30 different paediatric sub-specialties including post-acute burns care, cardiology, diabetes and neurology (Figure 1).

### Service costs

The fixed and variable costs of providing the telepaediatric service to Mackay and Hervey Bay during the first five years are shown in Table 1. Table 1 also compares the cost of telepaediatrics to the cost of sending patients to Brisbane to see the specialist in the outpatient department. Patient travel expenses were the major component of this cost.

**Table 1:** Actual telepaediatric service costs and potential costs if patients travelled to Brisbane

**Expenditure**	**Telepaediatrics (A$)**	**Conventional outpatients (A$)**
FIXED COSTS		
Equipment (5-year total annuatized cost)	128,191	0
ISDN installation	2655	0
ISDN line rental	32,400	0
Coordinators salaries	475,000	0
***Sub total***	***638,246***	***0***
		
VARIABLE COSTS		
Telecommunications *ISDN Line charges*	65,400	0
Staff salaries:		
*RCH Consultants ($200 per h)*	109,000	109,000
*RCH Nursing/Allied Health ($50 per h)*	27,250	27,250
*RCH Admin Support ($30 per h)*	0	16,350
*Regional presenter ($150 per h)*	81,750	0
*Regional admin support ($30 per h)*	16,350	0
Patient travel subsidy scheme:		
*Travel*	0	1,391,670
*Accommodation*	0	8,994
Project costs	18,000	0
***Sub total***	***317,750***	***1,553,264***
**Total cost**	**955,996**	**1,553,264**

### Average and variable costs

The average cost per consultation for the telepaediatric service was A$638. The average cost of a consultation conducted in the outpatient department at the RCH was A$1036. The variable cost of a telepaediatric consultation was A$212. It was largely composed of the salaries of the clinical staff required for the delivery of clinical services. The variable cost per consultation for an outpatient appointment was the same as the average cost (A$1036) since there were no fixed costs (Table 2).

**Table 2:** Telepaediatric service costs and potential costs if patients travelled to Brisbane

	**Telepaediatrics (A$)**	**Conventional outpatients (A$)**
Total costs	955,996.00	1,553,264.00
Average cost per consultation	637.76	1036.20
Variable cost per consultation	211.97	1036.20

### Threshold

**Figure 2:**
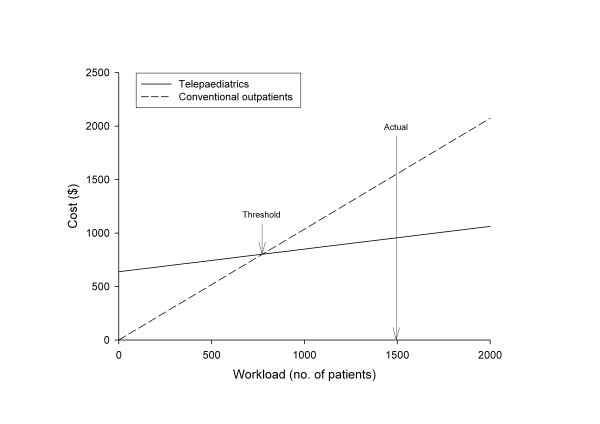
Total costs for telepaediatrics and conventional outpatients (A$).

The threshold point was reached at a workload of 774 consultations per five years (Figure 2).

The costs illustrated in Figure 2 show that the fixed costs of the telepaediatric service were about A$640,000, compared to zero fixed costs for the RCH outpatient service. At a workload of 1499 consultations, telepaediatrics would cost A$956,000 compared to A$1,553,000 for the conventional method. That is, there was a net saving of approximately A$600,000 for the actual workload of the present study.

### Sensitivity analysis

A sensitivity analysis was conducted to determine the effect of each of the cost components on the threshold. The change in threshold for a 1% change in the baseline value of the parameter of interest was calculated. This change was expressed as a ratio to the original baseline value. For example, in Table 3, the original value for the telepaediatric equipment was A$128,191 and the point of threshold was 774 patients. If the cost of videoconference equipment increased by 0.5%, the threshold was 775 patients. If the cost decreased by 0.5%, the threshold was 773 patients. That is, a change of 1% in the cost moved the threshold by 1.56 patients. This represents 0.20% of the baseline value of 774 patients.

**Table 3:** Changes to threshold (expressed as a ratio) when individual cost elements are adjusted by 1%

**A**	**B**	**C**	**D**	**E**	**F**	**G**	**H**
	Actual (A$)	Threshold (number of patients)	+ 0.5%	-0.5%	Change	Ratio (%)	Rank

**Telepaediatrics**							
Equipment (incl. interest)	128,191.01	774.36	775.14	773.58	1.56	0.20	3
ISDN installation	2655.00	774.36	774.37	774.34	0.03	0.00	9
ISDN line rental	32,400.00	774.36	774.56	774.16	0.39	0.05	6
Coordinators	475,000.00	774.36	777.24	771.48	5.76	0.74	2
ISDN call costs	65,400.00	774.36	774.56	774.15	0.41	0.05	6
RCH consultants	109,000.00	774.36	774.70	774.02	0.68	0.09	4
RCH nursing/allied health	27,250.00	774.36	774.44	774.27	0.17	0.02	7
Regional presenter	81,750.00	774.36	774.61	774.10	0.51	0.07	5
Regional admin support	16,350.00	774.36	774.41	774.31	0.10	0.01	8
Project costs	18,000.00	774.36	774.41	774.30	0.11	0.01	8
**Conventional outpatients**							
RCH consultants	109,000.00	774.36	774.70	774.02	0.68	0.09	4
RCH nursing/allied health	27,250.00	774.36	774.27	774.44	-0.17	-0.02	7
RCH admin support	16,350.00	774.36	774.31	774.41	-0.10	-0.01	8
Patient travel	1,391,670.00	774.36	770.02	778.74	-8.72	-1.13	1
Patient accommodation	8994.00	774.36	774.33	774.39	-0.06	-0.01	8
							
**Legend**							
Column A	Description of cost						
Column B	Original value of cost (in current evaluation)						
Column C	Original number of patients required to reach the point of threshold						
Column D	Adjusted threshold point when unit cost increased by 0.5%						
Column E	Adjusted threshold point when unit cost decreased by 0.5%						
Column F	Difference in threshold = (D-E)						
Column G	(Difference in threshold/original threshold) × 100						
Column H	Rank – most sensitive (1) to least sensitive (9) to change						

## Discussion

There is generally limited knowledge of the cost-effectiveness of telemedicine services compared to conventional methods of delivering health services [12-14]. The aim of the cost-analysis presented in this paper was to compare the costs of telepaediatrics to the costs of providing specialist outpatient services in the conventional manner, i.e. face to face. We suspect that the lack of quantitative evidence may be one of the reasons for the limited confidence in providing long-term funding for telemedicine services.

We examined the costs from the perspective of the health service provider. Several publications examining the economics of telemedicine applications in dermatology, ear, nose and throat (ENT) and radiology were used as a framework for this analysis [15-18]. The telepaediatric service has worked very well and has proven that the service is valuable for patients living in regional areas of Queensland [11]. The telepaediatric service was cheaper than the conventional method of sending patients to Brisbane for a specialist appointment at a workload exceeding 774 consultations. This is an encouraging result. In the present study 1499 consultations actually took place.

In addition to the economic savings generated by the telepaediatric service, there are other benefits of providing specialist paediatric services by telemedicine. The intangible benefits are often difficult to cost, but should be considered when evaluating the viability of such a service. We know from previous work that the telepaediatric service has made specialist services more easily accessible [11]. As a result, families spend less time travelling to their appointment, take less time off work, have less inconvenience and have lower out-of-pocket expenses than families who have to travel to the RCH [9].

One may argue that the staff requirements to do telepaediatrics are greater because a regional practitioner is in attendance during the consultation as well as the specialist. While this is true, there are other compensating factors to consider. For instance, the telepaediatric service ensures that collaboration takes place between the referring clinicians and specialists in Brisbane. Telepaediatric consultations give the regional staff a convenient method of accessing the opinion of a specialist whilst maintaining responsibility for the primary care of the patient. Consultations conducted via videoconference offer excellent learning opportunities for clinical teaching, collaboration and professional education. Arguably, these factors may also assist with recruitment and retention of medical staff in regional and remote hospitals.

In other telemedicine applications, staffing costs have been reduced by using a regional practitioner who is not a doctor. For example, in teleneurology, the medical officer was replaced by a specialist trained nurse [19]. The nurse maintains responsibility for organising the consultation at the remote site and for presenting the patient to the specialist. In Belfast, nurse-led consultations have been shown to be clinically feasible and an effective way of conducting telemedicine [20].

In its first years of operation, the telepaediatric service in Queensland has depended on research funding provided by the Commonwealth Government. In the longer term, it would seem sensible to use a proportion of the potential savings created by the telepaediatric service to cover operational costs.

A limitation of our study is that we estimated the costs of providing telepaediatric services to a group of patients during a defined timeframe and compared this with the potential cost of sending patients to the main tertiary hospital to see the specialist in person. It is unlikely that every consultation coordinated by the telepaediatric service represented a saved journey to Brisbane. However the exact number of patient transfers prevented remains unknown because the study was not a randomised control trial (RCT). Indeed, it is debatable whether a RCT would be possible for a range of ethical and practical reasons.

The ratio scores described in this paper demonstrate the relative importance of the elements in a cost analysis. In the present cost analysis, the threshold was most sensitive to changes in the costs of patient travel, followed by the costs of the coordinators' salaries and videoconferencing equipment. The costs that had the least impact on the threshold were the clinical staff salaries, ISDN installation, line rental and call costs, and accommodation. This information is important for planners of telemedicine services.

## Conclusion

The analysis presented in this paper compares the cost of providing a telepaediatric service to two selected hospitals in Queensland, to the potential costs if patients travel to Brisbane to see the specialist in person. The results show that considerable savings accrued to the health department, mainly due to reduced costs associated with patient travel. The results of the present study are relevant to health service planning in Queensland, and elsewhere in the world.

## Competing interests

The author(s) declare that they have no competing interests.

## Authors' contributions

AS and RW conceived the study and conducted the analysis. AS produced the first draft of the manuscript. PS provided valuable guidance with the interpretation of the economic evaluation. All authors contributed to the review and editing of the manuscript. All authors read and approved the final manuscript.

## Pre-publication history

The pre-publication history for this paper can be accessed here:


